# The SKIN-Q: An Innovative Patient-Reported Outcome Measure for Evaluating Minimally Invasive Skin Treatments for the Face and Body

**DOI:** 10.1089/fpsam.2023.0204

**Published:** 2024-06-06

**Authors:** Anne F. Klassen, Andrea L. Pusic, Manraj Kaur, Jasmine Mansouri, Elena Tsangaris, Steven Dayan, Jennifer Klok, Katie Armstrong, Katherine Santosa, Charlene Rae, Lotte Poulsen, Stefan J Cano

**Affiliations:** ^1^ Department of Pediatrics, McMaster University, Hamilton, Canada.; ^2^ Department of Surgery, Brigham and Women's Hospital, Harvard Medical School, Boston, Massachusetts, USA.; ^3^ Schulich School of Medicine and Dentistry, Western University, London, Canada.; ^4^ Dayan Facial Plastic Surgery, Chicago, Illinois, USA.; ^5^ SkinONE—Plastic Surgery, West Vancouver, Canada.; ^6^ McLean Clinic, Mississauga, Canada.; ^7^ The Center for Plastic Surgery at MetroDerm, Alpharetta, Georgia, USA.; ^8^ Research Unit for Plastic Surgery, Odense University Hospital, Løntoft, Nyhøj and Poulsen Plastic Surgery, Odense, Denmark.; ^9^ Modus Outcomes (a Division of Thread), Cheltenham, United Kingdom.

## Abstract

**Background:**

As the aesthetics field continues to innovate, it is important that outcomes are carefully evaluated.

**Objectives:**

To develop item libraries to measure how skin looks and feels from the patient perspective, that is, SKIN-Q.

**Methods:**

Concept elicitation interviews were conducted and data were used to draft the SKIN-Q, which was refined with patient and expert feedback. An online sample (i.e., Prolific) provided field-test data.

**Results:**

We conducted 26 qualitative interviews (88% women; 65% ≥ 40 years of age). A draft of the SKIN-Q item libraries were formed and revised with input from 12 experts, 11 patients, and 174 online participants who provided 180 survey responses. The psychometric sample of 657 participants (82% women; 36% aged ≥40 years) provided 713 completed surveys (facial, *n* = 595; body, *n* = 118). After removing 14 items, the psychometric analysis provided evidence of reliability (≥0.85) and validity for a 20-item set that measures how skin feels and a 46-item set that measures how skin looks. Short-form scales were tested to provide examples for how to utilize the item sets.

**Conclusion:**

The SKIN-Q represents an innovative way to measure satisfaction with skin (face and body) in the context of minimally invasive treatments.

**KEY POINTS**

**Question:** What outcomes matter to people seeking aesthetic skin treatments and how can we best measure these?

**Findings:** We identified what patients think is important about how their skin feels and looks and used what we learned to develop a new patient-reported outcome measure (i.e., the SKIN-Q). The SKIN-Q was tested in a large sample of people whose answers helped us identify the best subset of items to retain.

**Meaning:** The SKIN-Q can be used to measure how satisfied people are with how their skin looks and feels after a minimally invasive skin treatment that targets the face or body.

## Introduction

As aesthetic treatments continue to evolve, an increasing number of people are accessing an expanding range of aesthetic treatments to tighten, slim, reshape, and rejuvenate the skin on their face and body.^1^ To measure outcomes of aesthetic treatments, our research team developed patient-reported outcome measures (PROMs) for people having surgical and nonsurgical procedures. These PROMs include FACE-Q Aesthetics^2–9^ and BODY-Q.^10–12^ To measure outcomes specific to the skin, FACE-Q Aesthetics includes a 12-item scale that measures satisfaction with facial skin appearance. BODY-Q includes a 7-item scale that measures how bothered someone is by excess skin on their body.

Since FACE-Q Aesthetics and the BODY-Q were developed, PROM science has continued to evolve. While standard practice for PROM design involves the development of short forms composed of a limited set of items (i.e., questions), more recently, item libraries and item banks have been developed to provide a flexible approach that addresses a limitation of short forms, that is, they may not include the most important concepts for a specific patient population or context of use.^13,14^

To evaluate outcomes for aesthetic treatments, it is vital that PROMs have high content validity.^15–18^ To this end, we aimed to develop a comprehensive set of items to measure how skin feels and looks and provide a way to customize fit-for-purpose short-form scales. More specifically, subsets of items can be selected from an item set and scored by calibrating scores to the full set of items (i.e., item-bank approach^19,20^), or scored using estimates from independent samples (i.e., item-library approach).^21^ The short-form approach aims to reduce patient burden, while retaining reliable and valid measurement.

The specific aims of our study were as follows: (1) to elicit skin-related concepts important to patients having minimally invasive treatments that target the face and body; (2) to use the concepts to develop and refine a PROM (i.e., SKIN-Q); and (3) to determine how well the SKIN-Q performs psychometrically.

## Methods

Our study used a mixed-methods approach^22^ and followed internationally established guidelines for PROM development and validation.^15–18^ The study was coordinated at McMaster University (Canada). Ethics board approval (#13603) was obtained from the Hamilton Integrated Ethics Board (Canada).

An interpretative description qualitative approach was followed.^23^ Adult participants were recruited from six plastic surgery clinics located in Canada (three sites) and the USA (three sites) between October 22, 2021 and March 31, 2022. Clinic staff identified patients who varied by age, gender, race, and minimally invasive treatment. Patients who agreed to take part in the study provided written informed consent. Interviews took place by phone or on a secure web conferencing platform (i.e., Zoom) with experienced qualitative interviewers who followed an interview guide (Supplementary Data S1).

Interviews were audio-recorded, transcribed, and coded line-by-line. Coding was performed independently by two coders who achieved consensus on their initial set of codes. Codes were refined through constant comparison in Excel.^24^ Interviews continued until saturation of concepts was reached.^25^ Participants were provided a $100 USD gift card.

An item pool was developed and refined through a series of steps. In October 2022, participants from the qualitative interviews were invited to provide feedback online in REDCap.^26^ For each SKIN-Q item, participants selected one answer: (1) I do not understand the question; (2) I understand the question, but it could be worded better; (3) I understand the question, but it is not relevant to me; and (4) I understand the question and it is relevant to me. An open-text box was provided for suggestions. Participants were provided a $30 USD gift card.

Cognitive debriefing interviews were performed with survey participants using Zoom by an experienced interviewer. Participants were asked to provide feedback on the SKIN-Q, and to suggest missing content. Interviews were audio-recorded, transcribed, and analyzed. A gift card of $70 USD was provided. Experts in aesthetics, and representatives from the aesthetics industry, were emailed a copy of the SKIN-Q with instructions to point out items they thought were not relevant to patients and to suggest missing concepts.

Content validity was further explored using an online crowd-working platform, that is, Prolific (www.prolific.co). We conducted a screening survey in December 2022. At that time, residents of Canada and the USA fluent in English in the Prolific sample totaled 121,170. We paid participants the equivalent of 10.80 GBP per hour. We included participants who had the treatments listed in Supplementary Data S2 and excluded anyone who had *not* been to a plastic surgery or dermatology clinic for treatment in the past 12 months, and anyone who chose “none” or “other” for the treatment type, or “other for location of their body treatment.

For each item, participants chose one answer from the following: (1) I do not understand the question; (2) I understand the question, but it is NOT relevant to me, and (3) I understand the question and it is relevant to me. An open-text box was provided for suggestions. Data for scale refinement were downloaded into SPSS Version 28 (IBM Corporation, Armonk, NY, USA) for analysis.

A pilot field test was conducted using the Prolific sample described earlier. Survey invitations were sent in February 2023. A new Prolific sample was identified for the field-test study. The denominator for residents of Canada and the USA fluent in English for our screening survey in February 2023 was 121,448. Supplementary Data S2 inclusion criteria were used. We excluded participants who reported no treatment or chose “other” for the type of treatment, and for body treatments, anyone who chose “other” for treatment location and those whose treatment had worn off. Prolific participants were invited to complete a REDCap survey starting on March 3, 2023. At the end of the survey, participants were asked (yes/no) if they would be willing to complete the survey again in 7 days for a test–retest (TRT) study.

The pilot and field-test data were analyzed with Rasch Measurement Theory (RMT) analysis^27^ using RUMM2030 software^28^ and the unrestricted Rasch model for polytomous data. The pilot study was used to identify and remove items with extreme misfit to the Rasch model. For the field test, analysis was used to identify the best subset of items to retain for each item set based on a set of psychometric tests (Table 1).

**Table 1.** Psychometric tests performed

***Test***	***Description***
Thresholds for item response options	Examines whether the item response options measuring satisfaction are ordered on a continuum (e.g., a score of 1, on a continuum, should be lower than scores of 2 and higher). This approach is used to create a hierarchy of items to determine how items are ordered from easiest to hardest to endorse.
Item fit	Examines the extent to which observed data align with expected values based on the Rasch model. Item fit is assessed with fit residuals and chi-square statistics. Fit residuals summarize the observed and expected responses to an item by the sample and should ideally lie within the range −2.5 and +2.5. Chi-square values summarize the difference between observed and expected responses to an item for subgroups in the sample (class intervals) and should be nonsignificant after adjusting with the Bonferroni adjustment for multiple testing. Item characteristic curves can be viewed graphically.^29^ The sample was adjusted to 500 for tests of statistical fit.
Local dependency	Determines the extent of local dependency among items. Residual correlations were examined to identify any >0.30 above the average correlations. Items deemed locally dependent were included in subtests to determine their impact on scale reliability.^30^
Scale-to-sample targeting	Inspects the spread of person locations (i.e., satisfaction with skin in the sample) and item locations (i.e., range of satisfaction measured by the set of items). A scale that is better targeted has more coverage and has the mean person location close to the center of the items.^31^
DIF	Examines if items are invariant across subgroups. We examined DIF for age (i.e., 20–30, 31–40, >40), gender, and skin location (body and face). For the DIF analysis, we chose random samples with equal size samples in subgroups. For this analysis, we adjusted the sample size at 500 and chose random samples of equal size from each subgroup. When DIF was identified, variables were split for the relevant items, with both original and split person locations correlated to examine the impact of DIF on scale scoring.^32^
Reliability	Examines the accuracy of scores for a scale. Reliability statistics range from 0 to 1 with higher scores indicating greater reliability; scores should be >0.70.^33,34^ We explore three types of reliability: 1. Person separation index (PSI): In RMT, this test determines the extent to which people in the sample are separated by the items.^35^ 2. Cronbach alpha: In Rumm2030, we computed this statistic to measure internal reliability. 3. TRT reliability: A subset of participants completed the survey twice. We excluded anyone who reported an important change in satisfaction with how the skin feels or looks or who completed the TRT outside of 7–14 days. ICC with a two-way random effects model were computed using the transformed scores described hereunder. The ICC for how the skin looks was conducted separately for the body and face samples due to systematic missing data for face-specific items not completed by participants who had a body treatment.
Construct validity	Examines the extent to which the scale accurately measures what it purports to measure. Rasch logit scores were transformed into 0 (worse) to 100 (best), and short-form scores were calibrated using the item-bank approach. Parametric or nonparametric tests were used depending on the distribution of the data. Statistical significance was set at a two-tailed *p-*value of <0.05. We tested the following four hypotheses using the Rasch transformed scores: 1. SKIN-Q scores would be incrementally lower based on how much participants' aesthetic treatment had worn off (i.e., not at all, partially, and fully). 2. SKIN-Q scores would be incrementally lower based on how much (not at all, a little, moderately, very, and extremely) participants were bothered by lax or loose skin. 3. SKIN-Q scores would be incrementally lower based on how deep (none, mild, moderate, and severe/very severe) participants' Merz photo-numeric scores were for dynamic (i.e., crow's feet, forehead lines, and glabellar lines) and static (i.e., nasolabial folds, marionette lines, and lip) lines.^36^ 4. SKIN-Q scores would be lowest for participants who thought they looked older, and highest for participants who thought they looked younger than their age on the FACE-Q Aesthetics Age Visual Analog Scale.^4^ This scale asks how many years (i.e., ±15) younger or older people they think they look compared with their actual age. Answers were rescored into three groups: look younger, look age, and look older.

DIF, differential item functioning; ICC, intraclass correlation coefficients; RMT, Rasch Measurement Theory; TRT, test–retest.

## Results

Tables 2 and 3 show patient and treatment characteristics. The 26 participants from the qualitative sample had one or more minimally invasive facial treatments, and six participants had one or more minimally invasive body treatments involving the abdomen, chest, and thighs. Coding and analysis identified skin-specific concepts that were developed into items measuring how *skin looks* and how *skin feels.*

**Table 2.** Participant characteristics

	***Qualitative sample***	***Prolific***			
		***Cognitive sample***		***Psychometric sample***	
	***N* * = 26***	***N* * = 174***	***%***	***N* * = 657***	***%***
Sample					
Body only	0	45	25.9	62	9.5
Face only	26	123	70.7	539	82.0
Face and body	6	6	3.4	56	8.5
Country					
Canada	6	31	17.8	105	16.0
USA	20	143	82.2	550	83.7
Missing	0	0	0.0	2	0.3
Age					
20–29	3	44	25.3	200	30.4
30–39	6	46	26.4	218	33.2
40–49	7	30	17.2	112	17.1
50–59	6	35	20.1	81	12.3
≥60	4	19	10.9	46	7.0
Gender					
Woman	23	142	81.6	537	81.7
Man	3	29	16.7	108	16.4
Gender diverse	0	3	1.7	9	1.4
Prefer to not answer	0	0	0.0	2	0.3
Race					
White	22	127	73.0	443	67.4
Black	2	15	8.6	45	6.8
Latin American	0	15	8.6	33	5.0
East Asian	0	12	6.9	41	6.2
Middle Eastern	0	5	2.9	9	1.4
South Asian	1	4	2.3	15	2.3
Southeast Asian	1	4	2.3	11	1.7
Indigenous	0	1	0.6	2	0.3
Mixed race	0	0	0.0	54	8.2
Other	0	1	0.6	4	0.6
Marital status					
Married/common law	16	78	44.8	310	47.2
Single	7	61	35.1	266	40.5
Divorced	2	26	14.9	61	9.3
Separated	0	3	1.7	7	1.1
Widowed	1	2	1.1	3	0.5
Other/prefer not to answer	0	4	2.3	10	1.6
Fitzpatrick skin type					
Always burn and never tan	2	9	5.2	50	7.6
Usually burn and minimally tan	9	45	25.9	163	24.8
Mild burn and then tan	9	64	36.8	229	34.9
Rarely burn and always tan	4	33	19.0	144	21.9
Rarely burn and tan very easily	1	15	8.6	58	8.8
Never burn and never tan	1	8	4.6	13	2.0
Highest education					
Some high school	0	2	1.1	3	0.5
High school	1	11	6.3	35	5.3
Some college, trade, or university	4	24	13.8	102	15.5
College, trade or university degree	9	98	56.3	333	50.7
Some masters or doctoral degree	0	7	4.0	42	6.4
Masters or doctoral degree	11	31	17.8	141	21.5
Missing/prefer to not answer	1	1	0.6	1	0.2

**Table 3.** Treatment history reported by the qualitative sample and prolific participants based on number of surveys completed

		***Qualitative sample***	***Prolific***			
			***Cognitive sample***		***Psychometric sample***	
		***N* * = 26***	***N* * = 180***	***%***	*N* = 657^*^	***%***
Facial treatments						
Injectable	Botox	18	76	42.2	240	36.5
	Filler	17	71	39.4	167	25.4
	PRP	1	7	3.9	25	3.8
	Skin Booster	0	0	0.0	23	3.5
Skin resurfacing	Microdermabrasion	7	59	32.8	240	36.5
	Chemical peel	16	51	28.3	260	39.6
	Hydrafacial	2	40	22.2	280	42.6
	Laser	14	37	20.6	120	18.3
	Microneedling	2	30	16.7	148	22.5
	Light therapy	14	25	13.9	94	14.3
Skin tightening	Radio frequency	7	11	6.1	61	9.3
	High-intensity ultrasound	0	9	5.0	49	7.5
	Thread lift	1	6	3.3	29	4.4
Fat removal	Fat removal	1	6	3.3	29	4.4
Body treatments						
Injectables	Filler	0	17	9.4	41	6.2
	Skin Booster	0	0	0.0	18	2.7
Skin resurfacing	Laser	1	0	0.0	0	0.0
Fat reduction	Fat removal	0	6	3.3	22	3.3
	Cryolipolysis	2	28	15.6	47	7.1
	Laser lipolysis	0	10	5.6	20	3.0
	Radio frequency	1	8	4.4	15	2.3
	High-intensity focused electromagnetic	4	0	0.0	0	0.0
Skin tightening	High-intensity ultrasound	0	13	7.2	38	5.8
	Radio frequency	2	14	7.8	29	4.4
	Intense pulsed light and radio frequency	0	8	4.4	20	3.0
Cellulite	Cellulite treatment	0	17	9.4	35	5.3

^*^ Number of unique participants.

PRP, platelet-rich plasma.

Supplementary Data S3a,b shows item-level changes made after each round of patient/expert input. In Round 1, 11 of the 26 participants completed the 79 items survey providing 869 ratings. Of these, 0.1% of ratings were “I do not understand”; 10.9% of ratings were “I understand this question, but it could be worded better; 9.7% of ratings were “I understand this question, but it is not relevant to me”; and 79.3% of ratings were “I understand this question and it is relevant to me.”

Seven of the 11 participants took part in a cognitive debriefing interview. Round 1 also included three aesthetic plastic surgeons and one plastic surgery resident from Canada. Based on feedback, 67 items were retained, 7 were revised, 5 items were dropped, and 6 items were added resulting in a total of 80 items.

Round 2 included five plastic surgeons, one dermatologist, and two industry experts from Denmark, Canada, Sweden, and the USA. Based on this round, 20 items were retained, 58 items were revised, 2 items were dropped, and 8 items were added. At this point, the word “facial” was removed from all appearance items so that they were applicable to the face and body. At the end of Round 2, there were 86 items.

In Round 3, 939 Prolific participants accessed the screening survey. We invited the 281 people who met the study criteria to complete the survey. After exclusions, of the 174 respondents, 6 had both face and body treatments providing 180 (face = 129; body = 51) survey responses (Tables 2 and 3). Results for item comprehension and relevance are shown in Supplementary Data S3a,b and summarized in Supplementary Data S4. For the 86 items, the option “I do not understand the question” was chosen 1.3% of times, and the option “I understand the question and it is relevant to me” was chosen 74.6% of times. Based on this round, 79 items were retained, 2 items were revised, and 5 items were dropped. The pilot field test included 81 items.

The 174 participants were invited to complete the pilot field test and 161 respondents provided 167 assessments: 123 had facial treatments, and 44 had body treatments. Based on the RMT analysis, one skin item with poor fit was dropped. The field-test version had 80 items, that is, 58 measuring the skin looks (17 face-specific) and 22 measuring how the skin feels.

For the field test, we screened 2500 Prolific participants. After removing duplicates and incompletes, 2419 remained of which 904 met the inclusion criteria (face = 878; body = 157; both = 137). Of the 702 responses, 66 were incomplete, 51 had no treatment, 32 answered “other” for type of treatment, and 7 provided unreliable answers. The field-test sample included 546 surveys (face = 472; body = 74) from 496 participants.

For the RMT analysis, pilot (*N* = 167) and field-test (*N* = 546) data for the 657 participants were combined (total surveys = 713). Tables 2 and 3 show participant characteristics and treatment history. The 118 surveys from participants who had a body treatment, covered the following locations: abdomen = 60, thighs = 40, buttocks = 33, hips = 25, chest = 23, arms = 22, and lower legs = 8. RMT results are shown in Table 4 (scale-level) and Supplementary Data S5 (item-level).

**Table 4.** Rasch measurement theory scale-level statistics and other psychometric results

***Domain***	***Scale***	***RMT analysis***											***TRT Reliability***			
		***Items,* *N***	***Sample,* *N***	***RMT,* *N***	***Score on scale %***	**χ** ^***2***^	***DF***	***p***	***PSI***		**α**		***N***	***ICC***	***95% CI***	
									***+extr***	−****extr****	***+extr***	−****extr****			***LB***	***UB***
Looks	Item Library	46	713	690	96.8	463.10	414	0.05	0.98	0.98	0.99	0.98	152	0.87	0.82	0.90
	Skin Rejuvenation	9	713	663	93.0	68.17	54	0.09	0.94	0.93	0.96	0.94	155	0.86	0.81	0.90
	Skin Quality	12	713	677	95.0	133.10	108	0.05	0.93	0.92	0.95	0.93	155	0.88	0.83	0.91
	Facial Movement	7	595	528	88.7	47.44	35	0.08	0.89	0.87	0.93	0.90	108	0.81	0.73	0.87
Feels	Item Library	20	713	666	93.4	196.89	160	0.03	0.95	0.95	0.97	0.96	154	0.85	0.79	0.89
	Skin Rejuvenation	6	713	624	87.5	42.25	30	0.07	0.89	0.87	0.94	0.90	154	0.82	0.75	0.87
	Skin Quality	9	713	654	92.7	76.08	63	0.13	0.90	0.88	0.93	0.90	154	0.84	0.78	0.88

*α*, Cronbach alpha; *χ*^2^, chi-square; CI, confidence intervals; DF, degrees of freedom; PSI, person separation index; +extr, with extremes; −extr, without extremes; ICC, intraclass correlation coefficient; LB, lower bound; UB, upper bound; RMT, Rasch Measurement Theory.

For the 58 items measuring how the skin looks, 12 items were dropped due to poor item fit to the Rasch model. Of the remaining 46 items, all are relevant to facial skin and 33 are relevant to body skin. The 46 items had ordered thresholds (Supplementary Data S6a). After the Bonferroni adjustment, all items fit the Rasch model with nonsignificant chi-square *p*-values, and most (i.e., 28/46) had fit residuals ±2.5 or less. Differential item functioning (DIF) was identified for 19 items, with stable DIF (i.e., evident in all three random samples) only evident for four items in the age-group analysis. Pearson correlations between person locations for items before and after item split for DIF showed negligible impact on the scoring, that is, all correlations 1.000.

Most of the sample scored on the scale (96.8%). Data fit the Rasch model. Reliability was high with Person Separation Index (PSI) and Cronbach alpha values ≥0.98. A total of 30 pairs of items evidenced local dependency with residual correlations >0.30. PSI dropped 0.01 and Cronbach alpha dropped 0.05 after a subtest was performed. Supplementary Data S6b shows the Person-Item Threshold Distribution. The samples (face and body) were targeted to the scale. The floor (0.3) and ceiling (1.7) effects were low.

For the 22 items measuring how the skin feels, 2 items were dropped due to poor item fit to the Rasch model. The remaining 20 items are relevant to face and body. These items had ordered thresholds (Supplementary Data S7a), fit the Rasch model with nonsignificant chi-square *p*-values, and most (i.e., 13/20) had fit residuals ±2.5 or less. DIF was identified for 13 items, with stable DIF evident for 8 items in age group. Pearson correlations between person locations for items before and after item split for the specific items that evidenced DIF showed negligible impact on scoring, that is, correlations ≥0.994. Most of the sample scored on the scale (93.4%).

Data had slight misfit to the Rasch model (chi-square = 196.89, df = 160, *p* = 0.03). Most of the sample scored on the scale (93.4%). Reliability was high with PSI and Cronbach alpha values ≥0.95. A total of seven pairs of items evidenced local dependency. The impact on the reliability statistics was marginal, with a drop in the PSI values of 0.01 and Cronbach alphas as 0.06 after subtests were performed. Supplementary Data S7b shows the Person-Item Threshold Distribution. The samples (face and body) were targeted to the scale. Floor (0.8) and ceiling (5.9) effects were low.

Five example short-form scales were created: Skin Rejuvenation, Skin Quality, and Facial Movement. All items in Skin Rejuvenation and Skin Quality are relevant to both facial and body skin. Psychometric results are shown in Table 4. Data fit the Rasch model for the five short forms. All 43 items had ordered thresholds and nonsignificant *p*-values after Bonferroni adjustment. Fit residuals for 25/43 items were ±2.5 or less. Reliability was high with PSI and Cronbach alpha values ≥0.87. Items in two short-form scales evidenced local dependency. Subtests performed led to drops of ≤0.11 in reliability statistics, with all values ≥0.83. The short forms were well targeted; ≥87.5% of the sample scored on the scales' range of measurement.

**Fig. 1.**
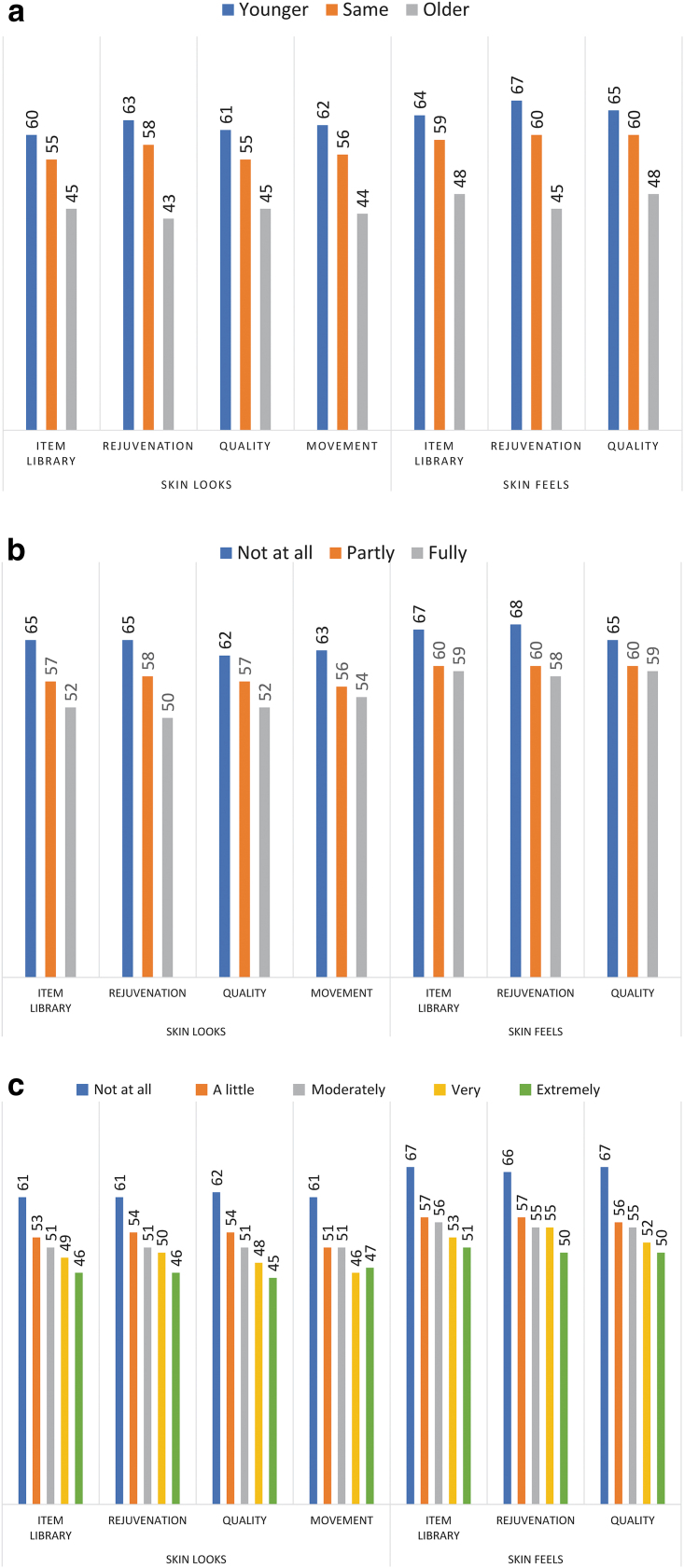
Mean scale scores for construct validation hypotheses based on the following questions: **(a)** How many years younger or older do you think you look compared with your actual age? All *p*-values <0.001; **(b)** How much have the effects of your most recent aesthetic treatment(s) worn off at this point? All *p*-values <0.001, except for Skin Feels for the Item Library *p* = 0.009 and for Quality *p* = 0.028; **(c)** How bothered are you by any lax or loose skin? All *p*-values <0.001.

Figure 1 shows the construct validity results. As hypothesized, SKIN-Q scores were incrementally lower the more treatment was reported as having worn off, being more bothered by lax skin, and looking older than one's actual age. Our hypothesis that SKIN-Q scores would be incrementally lower for deeper dynamic and static lines was also supported (Supplementary Data S8).

Table 4 shows the TRT results. We excluded one participant who completed the TRT on day 15 and 20 participants who reported change. Intraclass correlation coefficients results were ≥0.81.

## Discussion

We followed best practice guidelines for PROM development to create content to measure outcomes of skin treatments for the face and body. Our qualitative efforts elicited a set of skin-related concepts important to participants, which they deemed were comprehensive, relevant, and easy to understand. When tested in a large sample of people who had a minimally invasive treatment, the psychometric evidence supported the reliability and validity of the SKIN-Q item sets and five example short forms.

These findings add to the published literature on PROMs for aesthetic treatments by providing a means to measure satisfaction with how skin *feels* and *looks* anywhere on the face or body. The BODY-Q skin scale differs from SKIN-Q in that the BODY-Q measures how *bothered* people are by excess skin, an important concept in the context of body contouring after massive weight loss.^10,11^

The SKIN-Q has some conceptual overlap with the FACE-Q Aesthetics 12-item skin scale.^8^ Both scales measure satisfaction with skin. However, the FACE-Q version includes five unique items, and the word “facial” in each item. It is important to note that the FACE-Q Aesthetics skin scale is qualified as Medical Device Development Tools (MDDT) by the US Food and Drug Administration for use as a co-primary or secondary endpoint in clinical trials.^37^ The SKIN-Q, in contrast, is new and has not undergone similar qualification.

As aesthetic treatments continue to expand, it is vital that PROM development keeps pace. PRO item sets used as banks or libraries provide a flexible approach that addresses limitations inherent in short forms. In the choice of which PROM to use, potential users would be wise to maximize content validity and ensure the PROM is fit for purpose. Recommendations on the use of item libraries has been published.^13^

Our team has created the first available item sets for measuring satisfaction with skin in the context of minimally invasive aesthetic treatments. When used in clinical trials, these item sets can provide clinicians and researchers with the opportunity to pick a set of items relevant to measuring outcomes for their procedure or product. Short forms can either be calibrated in relation to the full set of items using the Rasch model (i.e., item bank approach), or stand-alone scoring can be created (i.e., item library approach).

These findings must be interpreted in the context of the study design. Limitations of our study include that the sample had fewer participants who underwent an aesthetic treatment for the body. Second, some treatments in our sample were represented by a small number of participants, and surgical treatments were not included. Third, our sample included only English-speaking people in Canada and the USA. Fourth, the treatment and clinical data were self-report, and was not verified clinically. Finally, the online platforms includes participants who self-select to take part and are paid for their involvement. There is evidence that the data provided through Prolific are high quality compared with other platforms.^38,39^

To conclude, the SKIN-Q is an innovative PROM that can be used to measure outcomes for minimally invasive treatments that target the face or body skin. The design of SKIN-Q makes it possible for end users to customize fit-for-purpose short-form scales to maximize content validity and reduce patient burden. Future research should examine psychometric properties not addressed in this article, such as responsiveness and minimally important differences. SKIN-Q can be accessed via www.qportfolio.org.

## References

[1] American Society of Plastic Surgeons. Plastic surgery statistics. Arlington Heights, IL; 2023. https://www.plasticsurgery.org/news/plastic-surgery-statistics?sub=2009+Plastic+Surgery+Statistics. Accessed June 20, 2023.

[2] Klassen AF, Cano SJ, Scott A, Snell L, Pusic AL. Measuring patient-reported outcomes in facial aesthetic patients: development of the FACE-Q. Facial Plast Surg. 2010;26(4):303–309. 20665408 10.1055/s-0030-1262313

[3] Pusic AL, Klassen AF, Scott AM, Cano SJ. Development and psychometric evaluation of the FACE-Q satisfaction with appearance scale: a new patient-reported outcome instrument for facial aesthetics patients. Clin Plast Surg. 2013;40(2):249–260. 23506765 10.1016/j.cps.2012.12.001

[4] Panchapakesan V, Klassen AF, Cano SJ, Scott AM, Pusic AL. Development and psychometric evaluation of the FACE-Q Aging Appraisal Scale and Patient-Perceived Age Visual Analog Scale. Aesthet Surg J. 2013;*33*(8):1099–1109. 24243890 10.1177/1090820X13510170

[5] Klassen AF, Cano SJ, Scott AM, Pusic AL. Measuring outcomes that matter to face-lift patients: development and validation of FACE-Q appearance appraisal scales and adverse effects checklist for the lower face and neck. Plast Reconstr Surg. 2014;133(1):21–30. 10.1097/01.prs.0000436814.11462.94 24105086

[6] Klassen AF, Cano SJ, Schwitzer JA, Scott AM, Pusic AL. FACE-Q scales for health-related quality of life, early life impact, satisfaction with outcomes, and decision to have treatment: development and validation. Plast Reconstr Surg. 2015;135(2):375–386. 25626785 10.1097/PRS.0000000000000895

[7] Klassen AF, Cano SJ, East CA, et al. Development and psychometric evaluation of the FACE-Q scales for patients undergoing rhinoplasty. JAMA Facial Plast Surg. 2016;*18*(1):27–35. 26605889 10.1001/jamafacial.2015.1445 PMC4831065

[8] Klassen AF, Cano SJ, Schwitzer JA, et al. Development and psychometric validation of the FACE-Q skin, lips, and facial rhytids appearance scales and adverse effects checklists for cosmetic procedures. JAMA Dermatol. 2016;152(4):443–451. 26934294 10.1001/jamadermatol.2016.0018 PMC4833666

[9] Klassen AF, Cano SJ, Grotting JC, et al. FACE-Q Eye Module for measuring patient-reported outcomes following cosmetic eye treatments. JAMA Facial Plast Surg. 2017;19(1):7–14. 27631534 10.1001/jamafacial.2016.1018 PMC5247311

[10] Klassen AF, Cano SJ, Scott A, Tsangaris E, Johnson J. Assessing outcomes in body contouring. Clin Plast Surg. 2014;1(4):645–654. 10.1016/j.cps.2014.06.004 25283452

[11] Klassen AF, Cano SJ, Soldin M, et al. The BODY-Q: a patient-reported outcome instrument for weight loss and body contouring treatments. Plast Reconstr Surg Glob Open. 2016;4(4):e694. 27200241 10.1097/GOX.0000000000000665 PMC4859238

[12] De Vries CEE, Klassen AF, Hoogbergen MM, Alderman AK, Pusic AL. Measuring outcomes in cosmetic abdominoplasty: the BODY-Q. Clin Plast Surg. 2020;47(3):429–436. 32448479 10.1016/j.cps.2020.03.003

[13] Piccinin C, Basch E, Bhatnagar V, et al. Recommendations on the use of item libraries for patient-reported outcome measurement in oncology trials: findings from an international, multidisciplinary working group. Lancet Oncol. 2023;24(2):e86–e95; doi: 10.1016/S1470-2045(22)00654-4 36725153

[14] Rose M, Bjorner JB, Gandek B, Bruce B, Fries JF, Ware JE. The PROMIS Physical Function item bank was calibrated to a standardized metric and shown to improve measurement efficiency. J Clin Epidemiol. 2014;67(5):516–526. 24698295 10.1016/j.jclinepi.2013.10.024 PMC4465404

[15] Food and Drug Administration. Guidance for industry patient-reported outcome measures: use in medical product development to support labeling claims. Silver Spring, MD; 2009. https://www.fda.gov/downloads/drugs/guidances/ucm193282.pdf. Accessed June 20, 2023.

[16] Terwee CB, Prinsen CA, Chiarotto A, et al. COSMIN methodology for assessing the content validity of PROMs. Amsterdam: VU University Medical Center; 2018. https://cosmin.nl/wp-content/uploads/COSMIN-methodology-for-content-validity-user-manual-v1.pdf. Accessed June 20, 2023.

[17] Patrick DL, Burke LB, Gwaltney CJ, et al. Content validity—establishing and reporting the evidence in newly developed patient-reported outcomes (PRO) instruments for medical product evaluation: ISPOR PRO good research practices task force report: part 1—eliciting concepts for a new PRO instrument. Value Health. 2011;14(8):967–977. 22152165 10.1016/j.jval.2011.06.014

[18] Patrick DL, Burke LB, Gwaltney CJ, et al. Content validity—establishing and reporting the evidence in newly developed patient-reported outcomes (PRO) instruments for medical product evaluation: ISPOR PRO Good Research Practices Task Force report: part 2—assessing respondent understanding. Value Health. 2011;14(8):978–988. 22152166 10.1016/j.jval.2011.06.013

[19] Hoppin B. Item banking using sample free calibration. Nature. 1968;219(5156):870872. 10.1038/219870a0 5673356

[20] Massof RW, Ahmadian L, Grover LL, et al. The Activity Inventory: an adaptive visual function questionnaire. Optom Vis Sci. 2007;84(8):763–774. 17700339 10.1097/OPX.0b013e3181339efd PMC6742517

[21] Regnault A, Pompilus F, Ciesluk A, et al. Measuring patient-reported physical functioning and fatigue in myelodysplastic syndromes using a modular approach based on EORTC QLQ-C30. J Patient Rep Outcomes. 202120;5(1):60. 34283303 10.1186/s41687-021-00334-w PMC8292469

[22] Regnault A, Willgoss T, Barbic S; International Society for Quality of Life Research Mixed Methods Special Interest Group. Towards the use of mixed methods inquiry as best practice in health outcomes research. J Patient Rep Outcomes. 2018;2(1):1–4. 29757311 10.1186/s41687-018-0043-8 PMC5934918

[23] Thorne S, Kirkham SR, MacDonald-Emes J. Interpretive description: a noncategorical qualitative alternative for developing nursing knowledge. Res Nurs Health. 1997;20(2):169–177. 9100747 10.1002/(sici)1098-240x(199704)20:2<169::aid-nur9>3.0.co;2-i

[24] Pope C, Ziebland S, Mays N. Qualitative research in health care. Analysing qualitative data. Br Med J. 2000;320(7227):114–116. 10625273 10.1136/bmj.320.7227.114 PMC1117368

[25] Sandelowski M. Theoretical saturation. In: Given LM, ed. The Sage Encyclopedia of Qualitative Methods. Vol. 1. Thousand Oaks, CA: Sage; 2008:875–876.

[26] Harris PA, Taylor R, Thielke R, Payne J, et al. Research electronic data capture (REDCap)—a metadata-driven methodology and workflow process for providing translational research informatics support. J Biomed Inform. 2009;42(2):377–381. 18929686 10.1016/j.jbi.2008.08.010 PMC2700030

[27] Rasch G. Probabilistic models for some intelligence and attainment tests. Copenhagen, Danish Institute for Education Research (Expanded edition 1980 with foreword and afterword by B.D. Wright, Chicago: The University of Chicago Press, 1980. 1960. Reprinted Chicago: MESA Press; 1993.

[28] Andrich D, Sheridan BS, Luo G. RUMM2030Plus: Rasch Unidimensional Models for Measurement. Perth, Western Australia: RUMM Laboratory; 2021.

[29] Hobart J, Cano S. Improving the evaluation of therapeutic interventions in multiple sclerosis: the role of new psychometric methods. Health Technol Assess. 2009;13(12):iii, ix-x, 1–177; doi: 10.3310/hta13120 19216837

[30] Christensen KB, Makransky G, Horton M. Critical values for Yen's Q 3: identification of local dependence in the rasch model using residual correlations. Appl Psychol Meas. 2017;41(3):178–194. 29881087 10.1177/0146621616677520 PMC5978551

[31] Cleanthous S, Bongardt S, Marquis P, Stach C, Cano S, Morel T. Psychometric analysis from EMBODY1 and 2 clinical trials to help select suitable fatigue pro scales for future systemic lupus erythematosus studies. Rheumatol Ther. 2021;8(3):1287–1301. 34244970 10.1007/s40744-021-00338-4 PMC8380611

[32] Andrich D, Hagquist C. Real and artificial differential item functioning. J Educ Behav Statist. 2012;37(3):387–416. 10.1177/0013164414534258 PMC5965593 29795818

[33] Nunnally JC. Psychometric Theory. 3rd ed. New York, NY: McGraw-Hill; 1994.

[34] Prinsen CA, Mokkink LB, Bouter LM, et al. COSMIN guideline for systematic reviews of patient-reported outcome measures. Qual Life Res. 2018;27:1147–1157. 29435801 10.1007/s11136-018-1798-3 PMC5891568

[35] Andrich D. An index of person separation in latent trait theory, the traditional KR.20 index, and the Guttman scale response pattern. Educ Res Perspect. 1982;9(1):95–104.

[36] Stella E, Di Petrillo A. Standard evaluation of the patient: the Merz Scale. In: Injections in Aesthetic Medicine: Atlas of Full-Face and Full-Body Treatment. Magda G, Rinna C, Enrica S. (eds.) Springer: Milan Heidelberg, New York, Dordrecht London; 2014; pp. 33–50.

[37] Food and Drug Administration. MDDT Summary of evidence and basis of qualification decision for FACE-Q| Aesthetics©. Silver Spring, MD; 2022. https://www.fda.gov/media/157956/download. Accessed June 20, 2023.

[38] Peer E, Rothschild D, Gordon A, et al. Data quality of platforms and panels for online behavioral research. Behav Res Methods. 2022;54(4):1643–1662. 34590289 10.3758/s13428-021-01694-3 PMC8480459

[39] Douglas BD, Ewell PJ, Brauer M. Data quality in online human-subjects research: Comparisons between MTurk, Prolific, CloudResearch, Qualtrics, and SONA. PLoS ONE. 2023;18(3):e0279720; doi: 10.1371/journal.pone.0279720 36917576 PMC10013894

