# Supplementary material for: The SKIN-Q: An Innovative Patient-Reported Outcome Measure for Evaluating Minimally Invasive Skin Treatments for the Face and Body
Source: Facial Plast Surg Aesthet Med. 2024 Jun 6;26(3):247–55. doi: 10.1089/fpsam.2023.0204 (PMC11295662; doi:10.1089/fpsam.2023.0204)
Supplement: Supplementary Data S1 [file fpsam.2023.0204_suppl_datas1.docx]

**S1: Qualitative interview topic guide**

| **TOPICS** | **QUESTIONS** |
| --- | --- |
| Treatments | Can you describe any cosmetic treatments you have had and plan to have?  What are/were your expectations for the treatment you have had/plan to have? |
| Face: Looks | Can you describe how your FACE LOOKED before treatment?  Can you describe any change in how your FACE LOOKED after treatment? |
| Skin: Looks | Can you describe how your SKIN (on face and body) LOOKED before treatment?  Can you describe any change in how your SKIN (on face and body) LOOKED after treatment? |
| Skin: Feels | Can you describe how your SKIN (on face and body) FELT before treatment?  Can you describe in detail any change in the how your SKIN (on face and body) FELT after treatment? |
| Facial Expressions Animation  Movement | Can you describe how your FACE LOOKED after treatment when you moved your face or showed expression?  Can you describe any change in your ability to MOVE your FACE or show expression after treatment? |
| Satisfaction & Importance | Can you describe the aspects of how your FACE/SKIN (on face and body) LOOKS that you are most and least satisfied with and which aspects are the most/least important to you?  Can you describe the aspects of how your FACE/SKIN (on face and body) FEELS that you are most and least satisfied with and which aspects are the most/least important to you? |
| Natural | Many people seeking antiaging treatments want to look NATURAL (not overdone or fake). Is this concept important to you?  How would you define natural?  How would you describe a natural look for the treatment you have had/plan to have? |
| Aging Appraisal | Can you describe how old/young you LOOKED before treatment and how it made you feel?  Can you describe how old/young you LOOKED after treatment and how it made you feel? |
| Psychological impact | Can you describe how your appearance affects how you FELT before treatment?  Can you any change in how you FELT after treatment? |
| Social impact | Can you describe how your appearance affects your interactions with people before treatment?  Can you describe how any change in your appearance affected your interactions with people after treatment? |
| Recovery from treatment | Can you describe the recovery process for the treatment you had? |
| Overall outcome | What is your overall opinion about the treatment you had? |
