# Supplementary material for: The SKIN-Q: An Innovative Patient-Reported Outcome Measure for Evaluating Minimally Invasive Skin Treatments for the Face and Body
Source: Facial Plast Surg Aesthet Med. 2024 Jun 6;26(3):247–55. doi: 10.1089/fpsam.2023.0204 (PMC11295662; doi:10.1089/fpsam.2023.0204)
Supplement: Supplementary Data S2 [file fpsam.2023.0204_suppl_datas2.docx]

**S2: Screening questions used in Prolific**

| AESTHETICS TREATMENTS - FACE | |
| --- | --- |
| In the PAST 12 MONTHS, have you been to a DERMATOLOGY or a PLASTIC SURGERY CLINIC to have a FACIAL AESTHETIC treatment? | 0, No  1, Yes |
| In the PAST 12 MONTHS, have you had any of these FACIAL AESTHETIC Treatments:  Choose all that apply | 0, NONE  1, BOTULINUM TOXIN A - ie, Botox, Dysport, Xeomin or Jeuveau, Xeomin  2, FILLER - eg, Restylane, Juvederm, Radiesse, Sculptra  3, FAT REDUCTION - eg, Kybella to treat a double chin  4, SKIN BOOSTER (eg, Prophilo) (*asked in field-test screen)  5, PLATELET RICH PLASMA (PRP) injections  6, SKIN TIGHTENING with ultrasound - eg, Ultherapy  7, SKIN TIGHTENING with Radio-frequency - eg, Thermage, Morpheus8, Exilis, Profound RF  8, CHEMICAL PEEL  9, MICRODERMABRASION  10, LASER - eg, CO2, Vbeam, Fraxel  11, INTENSE PULSED LIGHT Light (IPL) - eg, Lynton Lumina IPL  12, MICRONEEDLING  13, HYDRAFACIAL  14, THREADLIFT  15, Other |
| You said you had BOTOX injected. What was the MAIN REASON for having BOTOX? | 1, Cosmetic reasons - to look better, younger, refreshed  2, Medical reasons - to treat migraines, to stop grinding teeth  3, Other reason  88, None of the above |
| You said you a SOFT TISSUE FILLER injected. The last time you had filler, where was the filler injected?  Choose all that apply | 1, Cheeks - to add volume and restore fullness  2, Lips - to plump or to smooth out lip lines  3, Other |
| AESTHETICS TREATMENTS - BODY | |
| In the PAST 12 MONTHS, have you been to a DERMATOLOGY or a PLASTIC SURGERY CLINIC to have a COSMETIC treatment for your BODY? | 0, No  1, Yes |
| In the PAST 12 MONTHS, have you had any of these Cosmetic Treatments somewhere on your BODY (not your FACE): | 0, NONE  1, FILLER - eg, Restylane, Juvederm, Radiesse, Sculptra  2, FAT REMOVAL - eg, injection with Kybella  3, FAT REDUCTION with freezing - eg, Coolsculpting  4, FAT REDUCTION with laser - eg, Sculpsure  5, FAT REDUCTION with Radio-Frequency - eg, Vanquish  6, SKIN TIGHTENING with ultrasound - eg, Ultherapy  7, SKIN TIGHTENING with Radio-frequency - eg, Thermage, Morpheus8, Exilis, Profound RF  8, SKIN TIGHTENING with Intense Pulsed Light (IPL) and Radio-Frequency  9, CELLULITE treatment (eg, Cellulaze, Cellfina, QWO  10, Other |
| You said you a COSMETIC treatment for your BODY. For your MOST RECENT cosmetic treatment, what part of your BODY was treated?  Choose all that apply | 1, Arms  2, Hands  3, Upper Chest (ie, decolletage)  4, Chest  5, Abdomen  6, Hips  7, Buttocks  8, Thighs  9, Lower legs  10, Other |
