# Supplementary material for: The SKIN-Q: An Innovative Patient-Reported Outcome Measure for Evaluating Minimally Invasive Skin Treatments for the Face and Body
Source: Facial Plast Surg Aesthet Med. 2024 Jun 6;26(3):247–55. doi: 10.1089/fpsam.2023.0204 (PMC11295662; doi:10.1089/fpsam.2023.0204)
Supplement: Supplementary Data S3 [file fpsam.2023.0204_suppl_datas3.docx]

**S3a: Changes made to items in each round sorted by Prolific sample relevance ratings**

| SKIN LOOKS | | | | | | | | | | | | | |
| --- | --- | --- | --- | --- | --- | --- | --- | --- | --- | --- | --- | --- | --- |
| ROUND 1: PATIENTS AND EXPERTS | | **ROUND 2: EXPERTS** | | **ROUND 3: PROLIFIC** | | | | | | | **PILOT FIELD-TEST** | | **FT** |
|  |  |  |  |  | **N** | **UNDERSTAND NO** | | **RELEVANT YES** | |  |  |  |  |
|  |  |  |  |  |  | **N** | **%** | **N** | **%** |  |  |  |  |
| 1. How smooth your facial skin looks? | retain |  | revise | How smooth your skin looks? | 179 | 1 | 0.6 | 158 | 88.3 | retain |  | retain | retain |
| 1. How healthy your facial skin looks? | retain |  | revise | How healthy your skin looks? | 179 | 1 | 0.6 | 153 | 85.5 | retain |  | retain | retain |
| 1. The overall quality of your facial skin? | retain |  | revise | The overall quality of your skin? | 179 | 1 | 0.6 | 152 | 84.9 | retain |  | retain | retain |
| 1. How your facial skin looks when your face is relaxed? | retain |  | revise | How your skin looks when your face is relaxed? | 128 | 0 | 0 | 108 | 84.4 | retain |  | retain | retain |
| 1. How good your facial skin looks? | retain |  | revise | How good your skin looks? | 179 | 1 | 0.6 | 151 | 84.4 | retain |  | retain | retain |
| 1. How rejuvenated your facial skin looks? | retain |  | revise | How rejuvenated your skin looks? | 179 | 2 | 1.1 | 151 | 84.4 | retain |  | retain | retain |
| 1. How nice your facial skin looks? | retain |  | revise | How nice your skin looks? | 179 | 3 | 1.7 | 151 | 84.4 | retain |  | retain | drop |
| 1. How your facial skin looks up close? | retain |  | revise | How your skin looks up close? | 179 | 2 | 1.1 | 149 | 83.2 | retain |  | retain | retain |
| 1. How firm your facial skin looks? | retain |  | revise | How firm your skin looks? | 179 | 4 | 2.2 | 150 | 83.8 | retain |  | retain | retain |
| 1. How your facial skin looks in a mirror (straight on)? | retain |  | revise | How your skin looks in a mirror (straight on)? | 128 | 1 | 0.8 | 105 | 82.0 | retain |  | retain | drop |
| 1. The texture of your facial skin? | retain |  | revise | The texture of your skin? | 179 | 1 | 0.6 | 148 | 82.7 | retain |  | retain | retain |
| 1. How natural your facial skin looks? | retain |  | revise | How natural your skin looks? | 179 | 2 | 1.1 | 147 | 82.1 | retain |  | retain | retain |
| 1. How your facial skin looks without makeup? | retain |  | revise | How your skin looks without makeup? | 128 | 1 | 0.8 | 104 | 81.3 | retain |  | retain | drop |
| 1. How noticeable any lines (wrinkles) on your face are? | retain |  | revise | How noticeable any lines (wrinkles) in your skin are? | 179 | 1 | 0.6 | 145 | 81.0 | retain |  | retain | drop |
| 1. How fresh your facial skin looks? | retain |  | revise | How fresh your skin looks? | 179 | 3 | 1.7 | 144 | 80.4 | retain |  | retain | retain |
|  |  |  | add | How young your skin looks? | 128 | 2 | 1.6 | 103 | 80.5 | retain |  | retain | retain |
| 1. How hydrated your facial skin looks? | retain |  | revise | How hydrated your skin looks? | 179 | 0 | 0 | 142 | 79.3 | retain |  | retain | retain |
| 1. How radiant your facial skin looks? | retain |  | revise | How radiant your skin looks? | 179 | 0 | 0 | 142 | 79.3 | retain |  | retain | retain |
|  |  |  | add | How refreshed your skin looks? | 179 | 4 | 2.2 | 142 | 79.3 | retain |  | retain | drop |
| 1. How youthful your facial skin looks? | retain |  | revise | How youthful your skin looks? | 179 | 2 | 1.1 | 141 | 78.8 | retain |  | retain | retain |
| 1. How refreshed your facial skin makes you look? | retain |  | revise | How refreshed your skin makes you look? | 179 | 0 | 0 | 142 | 79.3 | retain |  | retain | retain |
| 1. How your facial skin looks in photos? | retain |  | revise | How your skin looks in photos? | 179 | 1 | 0.6 | 140 | 78.2 | retain |  | retain | retain |
| 1. How your facial skin looks compared with other people your age? | retain |  | revise | How your skin looks compared with other people your age? | 179 | 2 | 1.1 | 140 | 78.2 | retain |  | retain | drop |
|  |  |  | add | How relaxed your skin looks (not tight or pulled)? | 179 | 3 | 1.7 | 139 | 77.7 | retain |  | retain | retain |
|  | add | How your facial skin looks when you show expression? | revise | How your skin looks when you show expression? | 128 | 1 | 0.8 | 99 | 77.3 | retain |  | retain | retain |
| 1. How flawless your facial skin looks. | retain |  | revise | How flawless your skin looks. | 179 | 3 | 1.7 | 139 | 77.7 | retain |  | retain | retain |
| 1. How clear your facial skin looks (complexion)? | retain |  | revise | How clear your skin looks (complexion)? | 179 | 1 | 0.6 | 137 | 76.5 | retain |  | retain | drop |
| 1. How attractive your facial skin makes you look? | retain |  | revise | How attractive your skin makes you look? | 179 | 3 | 1.7 | 137 | 76.5 | retain |  | retain | retain |
| 1. How vibrant your facial skin looks? | retain |  | revise | How vibrant your skin looks? | 179 | 4 | 2.2 | 136 | 76.0 | retain |  | retain | retain |
| 1. How tight your facial skin looks? | revise | How tight your facial skin looks (not loose)? | revise | How tight your skin looks (not loose)? | 179 | 2 | 1.1 | 135 | 75.4 | retain |  | retain | retain |
| 1. How even-toned your facial skin looks? | retain |  | revise | How even-toned your skin looks? | 179 | 0 | 0 | 134 | 74.9 | retain |  | retain | retain |
| 1. How your facial skin looks when you raise your eyebrows? | retain |  | revise | How your skin looks when you raise your eyebrows? | 128 | 1 | 0.8 | 96 | 75.0 | retain |  | retain | retain |
| 1. How soft your facial skin looks? | retain |  | revise | How soft your skin looks? | 179 | 1 | 0.6 | 133 | 74.3 | retain |  | retain | retain |
| 1. How your facial skin looks from different angles? | retain |  | revise | How your skin looks from different angles? | 179 | 4 | 2.2 | 133 | 74.3 | retain |  | retain | retain |
| 1. How your facial skin looks when you smile? | retain |  | revise | How your skin looks when you smile? | 128 | 0 | 0 | 95 | 74.2 | retain |  | retain | retain |
| 1. How plump your facial skin looks? | revise | How full (ie, plump and youthful) your facial skin looks? | revise | How full (ie, plump and youthful) your skin looks? | 179 | 0 | 0 | 132 | 73.7 | retain |  | retain | retain |
| 1. How even-colored your facial skin looks? | retain |  | revise | How even-colored your skin looks? | 179 | 1 | 0.6 | 131 | 73.2 | retain |  | retain | drop |
| 1. The age your facial skin makes you look? | retain |  | revise | The age your skin makes you look? | 179 | 5 | 2.8 | 131 | 73.2 | retain |  | retain | retain |
| 1. How your facial skin looks when you look your best? | retain |  | revise | How your skin looks when you look your best? | 128 | 2 | 1.6 | 93 | 72.7 | retain |  | retain | retain |
| 1. How rested your facial skin makes you looks? | retain |  | revise | How rested your skin makes you looks? | 179 | 5 | 2.8 | 129 | 72.1 | retain |  | retain | retain |
| 1. How your facial skin looks when you laugh? | retain |  | revise | How your skin looks when you laugh? | 128 | 0 | 0 | 92 | 71.9 | retain |  | retain | retain |
| 1. How your facial skin looks when you frown? | retain |  | revise | How your skin looks when you frown? | 128 | 2 | 1.6 | 92 | 71.9 | retain |  | retain | retain |
| 1. How lifted your facial skin looks (not saggy)? | retain |  | revise | How lifted your skin looks (not saggy)? | 179 | 1 | 0.6 | 126 | 70.8 | retain |  | retain | retain |
| 1. How your pores look? | retain |  | revise | How your pores look? | 179 | 3 | 1.7 | 126 | 70.4 | retain |  | retain | drop |
| 1. How your facial skin looks from the side (your profile)? | retain |  | revise | How your skin looks from the side (your profile)? | 179 | 1 | 0.6 | 125 | 69.8 | retain |  | retain | retain |
| 1. The amount of elasticity in your facial skin? | retain |  | revise | The amount of elasticity in your skin? | 179 | 4 | 2.2 | 124 | 69.3 | retain |  | retain | retain |
| 1. How your facial skin looks when you squint? | retain |  | revise | How your skin looks when you squint? | 128 | 2 | 1.6 | 88 | 68.8 | retain |  | retain | retain |
| 1. How your facial skin looks with makeup? | retain |  | revise | How your skin looks with makeup? | 128 | 0 | 0 | 89 | 69.5 | retain |  | retain | retain |
| 1. How bright your facial skin looks? | retain |  | revise | How bright your skin looks? | 179 | 1 | 0.6 | 125 | 69.8 | retain |  | retain | retain |
| 1. How clean your facial skin looks? | retain |  | revise | How clean your skin looks? | 179 | 2 | 1.1 | 121 | 67.6 | retain |  | retain | drop |
| 1. How much your facial skin glows? | retain |  | revise | How much your skin glows? | 179 | 2 | 1.1 | 122 | 68.2 | retain |  | retain | retain |
| 1. How your facial skin looks at the end of your day? | retain |  | revise | How your skin looks at the end of your day? | 179 | 2 | 1.1 | 120 | 67.0 | retain |  | retain | drop |
| 1. How your facial skin looks when you are ready to go out? | retain |  | revise | How your skin looks when you are ready to go out? | 128 | 3 | 2.3 | 86 | 67.2 | retain |  | retain | retain |
| 1. How your facial skin looks under a bright light? | retain |  | revise | How your skin looks under a bright light? | 179 | 3 | 1.7 | 117 | 65.4 | retain |  | retain | retain |
| 1. How the tone (color) of your facial skin looks? | retain |  | revise | How the tone (color) of your skin looks? | 179 | 0 | 0 | 111 | 62.0 | retain |  | retain | retain |
|  | add | The thickness of your facial skin (not thin and fragile)? |  | The thickness of your skin (not thin and fragile)? | 179 | 8 | 4.5 | 101 | 56.4 | revise | How thick your skin looks (not thin or fragile)? | retain | retain |
| 1. How your facial skin looks when you first wake up? | retain |  | revise | How your skin looks when you first wake up? | 179 | 4 | 2.2 | 98 | 54.7 | retain |  | retain | drop |
| 1. How your facial skin looks when you see yourself on a screen (eg, Zoom, Facetime)? | revise | How your facial skin looks when on a screen (eg, Zoom, Facetime)? | revise | How your skin looks when on a screen (eg, Zoom, Facetime)? | 128 | 1 | 0.8 | 69 | 53.9 | retain |  | retain | retain |
| 1. How your facial skin looks in a photo that uses a filter? | retain |  | revise | How your skin looks in a photo that uses a filter? | 128 | 2 | 1.6 | 57 | 44.5 | retain |  | drop |  |
|  |  |  | add | How old your skin looks? | 179 | 5 | 2.8 | 132 | 73.7 | drop |  |  |  |
| 1. How old your facial skin makes you look? | retain |  | revise | How old your skin makes you look? | 179 | 3 | 1.7 | 130 | 72.6 | drop |  |  |  |
| 1. How your facial skin looks from far away? | retain |  | revise | How your skin looks from far away? | 179 | 1 | 0.6 | 122 | 68.2 | drop |  |  |  |
| 1. How your facial makeup goes on (smoothly)? | retain |  | drop |  |  |  |  |  |  |  |  |  |  |
| 1. The amount of facial makeup you need to wear? | retain |  | drop |  |  |  |  |  |  |  |  |  |  |
| 1. How much your facial skin shines (not dull)? | drop |  |  |  |  |  |  |  |  |  |  |  |  |
| 1. How your facial skin looks on a screen compared to in person (eg, Zoom, facebook)? | drop |  |  |  |  |  |  |  |  |  |  |  |  |
| 1. How flat any lines on your face look? | drop |  |  |  |  |  |  |  |  |  |  |  |  |

Sample of 128 indicates items that were tested in facial aesthetics patients only in the Round 3 Prolific survey. One person from the sample of 180 did not complete the Round 3 Prolific survey.

**S3b: Changes made to items in each round sorted by Prolific sample relevance ratings**

| SKIN FEELS | | | | | | | | | | | | | |
| --- | --- | --- | --- | --- | --- | --- | --- | --- | --- | --- | --- | --- | --- |
| ROUND 1: PATIENTS AND EXPERTS | | **ROUND 2: EXPERTS** | **ROUND 3: PROLIFIC** | | | | | | | | **PILOT**  **FIELD-TEST**  **FIELD** | | **FT** |
|  |  |  |  | | **N** | **UNDERSTAND NO** | | **RELEVANT YES** | |  |  |  |  |
|  |  |  |  |  |  | **N** | **%** | **N** | **%** |  |  |  |  |
| 1. How smooth your facial skin feels? | retain |  | retain |  | 179 | 1 | 0.6 | 157 | 87.7 | retain |  | retain | retain |
| 1. How healthy your facial skin feels? | retain |  | retain |  | 179 | 2 | 1.1 | 151 | 83.9 | retain |  | retain | retain |
| 1. How rejuvenated your facial skin feels? | retain |  | retain |  | 179 | 4 | 2.2 | 151 | 84.4 | retain |  | retain | retain |
| 1. How good your facial skin feels? | retain |  | retain |  | 179 | 5 | 2.8 | 145 | 81.0 | retain |  | retain | retain |
| 1. How firm your facial skin feels? | retain |  | retain |  | 179 | 1 | 0.6 | 144 | 80.4 | retain |  | retain | retain |
|  |  |  | add | How refreshed your facial skin feels? | 179 | 1 | 0.6 | 142 | 79.3 | retain |  | retain | retain |
| 1. How youthful your facial skin feels? | retain |  | retain |  | 179 | 1 | 0.6 | 141 | 78.8 | retain |  | retain | retain |
|  |  |  | add | How natural your skin feels? | 129 | 3 | 2.3 | 103 | 79.8 | retain |  | retain | retain |
| 1. How hydrated your facial skin feels? | revise | How hydrated your facial skin feels (not flaky or dry)? | retain |  | 179 | 1 | 0.6 | 140 | 78.2 | retain |  | retain | retain |
| 1. How moist your facial skin feels? | revise | How comfortable your facial skin feels (not too dry or tight)? | retain |  | 179 | 0 | 0 | 138 | 77.1 | retain |  | retain | retain |
|  | add | The texture of your facial skin? | retain |  | 179 | 0 | 0 | 138 | 77.1 | retain |  | retain | retain |
| 1. How tight your facial skin feels? | revise | How tight your facial skin feels (not loose)? | retain |  | 179 | 4 | 2.2 | 138 | 77.1 | retain |  | retain | retain |
| 1. How soft your facial skin feels? | retain |  | retain |  | 179 | 1 | 0.6 | 136 | 76.0 | retain |  | retain | retain |
| 1. How nice your facial skin feels? | retain |  | retain |  | 179 | 3 | 1.7 | 135 | 75.4 | retain |  | retain | drop |
| 1. How flawless your facial skin feels? | retain |  | retain |  | 179 | 2 | 1.1 | 134 | 74.9 | retain |  | retain | retain |
| 1. How clear your facial skin feels? | retain |  | retain |  | 179 | 2 | 1.1 | 132 | 73.7 | retain |  | retain | retain |
| 1. How plump your facial skin feels? | revise | How full (ie, plump and youthful) your facial skin feels`? | retain |  | 179 | 2 | 1.1 | 131 | 73.2 | retain |  | retain | retain |
|  | add | The amount of elasticity in your facial skin? | retain |  | 179 | 3 | 1.7 | 129 | 72.1 | retain |  | retain | retain |
| 1. The age your facial skin feels? | retain |  | retain |  | 179 | 6 | 3.4 | 127 | 70.9 | retain |  | retain | drop |
| 1. How clean your facial skin feels? | retain |  | retain |  | 179 | 3 | 1.7 | 125 | 69.8 | retain |  | retain | retain |
|  | add | How new your facial skin feels? | retain |  | 179 | 4 | 2.2 | 116 | 64.8 | retain |  | retain | retain |
|  | add | The thickness of your facial skin (not thin and fragile)? | retain |  | 179 | 9 | 5.1 | 91 | 51.1 | revise | How thick your skin feels (not thin or fragile)? | retain | retain |
|  |  |  | add | How young your skin feels? | 129 | 6 | 4.7 | 88 | 68.2 | drop |  | retain | retain |
|  |  |  | add | How old your facial skin feels? | 179 | 10 | 5.6 | 102 | 57.0 | drop |  |  |  |
| 1. How flat your facial skin feels (smooth)? | drop |  |  |  |  |  |  |  |  |  |  |  |  |
| 1. How even your facial skin feels? | drop |  |  |  |  |  |  |  |  |  |  |  |  |

Sample of 129 indicates items that were tested in facial aesthetics patients only in the Round 3 Prolific survey. One person from the sample of 180 did not complete the Round 3 Prolific survey.
