# Supplementary material for: The SKIN-Q: An Innovative Patient-Reported Outcome Measure for Evaluating Minimally Invasive Skin Treatments for the Face and Body
Source: Facial Plast Surg Aesthet Med. 2024 Jun 6;26(3):247–55. doi: 10.1089/fpsam.2023.0204 (PMC11295662; doi:10.1089/fpsam.2023.0204)
Supplement: Supplementary Data S4 [file fpsam.2023.0204_suppl_datas4.docx]

**S4: Summary results for understood and relevant**

| **Scale** | **Sample** | **# Raters** | **# Items** | **Raters x Items** | **Understood** | | **Relevant** | |
| --- | --- | --- | --- | --- | --- | --- | --- | --- |
|  |  |  |  |  | **N** | **%** | **N** | **%** |
| Skin – Feels | Face | 129 | 24 | 3096 | 3039 | 98.2 | 2352 | 76.0 |
|  | Body | 50 | 22 | 1100 | 1083 | 98.5 | 781 | 71.0 |
| Skin – Looks | Face | 128 | 62 | 7936 | 7862 | 99.1 | 6004 | 75.7 |
|  | Body | 51 | 47 | 2397 | 2350 | 98.0 | 1706 | 71.2 |
| Total | | 358 | 155 | 14529 | 14334 | 98.7 | 10843 | 74.6 |

The number of items tested varied by sample for the 2 scales.
