# Supplementary material for: The SKIN-Q: An Innovative Patient-Reported Outcome Measure for Evaluating Minimally Invasive Skin Treatments for the Face and Body
Source: Facial Plast Surg Aesthet Med. 2024 Jun 6;26(3):247–55. doi: 10.1089/fpsam.2023.0204 (PMC11295662; doi:10.1089/fpsam.2023.0204)
Supplement: Supplementary Data S5 [file fpsam.2023.0204_suppl_datas5.docx]

**S5: RMT item level fit statistics and differential item function results**

| **Scales** | **Item Fit Statistics** | | | | | | | **Differential Item Function*** | | | **Short Forms** | | |
| --- | --- | --- | --- | --- | --- | --- | --- | --- | --- | --- | --- | --- | --- |
| **SKIN LOOKS** | **Location** | **SE** | **Fit**  **Residual** | **DF** | **χ^2^** | **DF** | **p-value** | **Age** | **Gender** | **Body/Face** | **Facial Movement** | **Skin Rejuvenation** | **Skin Quality** |
| 1. Look best | -1.24 | 0.08 | 1.45 | 565.16 | 13.51 | 9 | 0.14 | - | - | - | - | - | - |
| 1. Natural | -1.12 | 0.07 | 0.28 | 671.19 | 13.88 | 9 | 0.13 | - | - | - | - | - | - |
| 1. Go out | -1.00 | 0.08 | 0.51 | 565.16 | 6.70 | 9 | 0.67 | - | - | - | - | - | - |
| 1. Relaxed look | -0.80 | 0.07 | 3.26 | 671.19 | 18.04 | 9 | 0.03 | - | 1,2 | - | - | - | - |
| 1. Smile | -0.73 | 0.08 | 0.88 | 565.16 | 6.02 | 9 | 0.74 | - | - | - | **YES** | - | - |
| 1. Thick | -0.66 | 0.07 | 2.68 | 671.19 | 7.14 | 9 | 0.62 | - | - | 1,2 | - | - | **YES** |
| 1. Laugh | -0.60 | 0.08 | 0.32 | 565.16 | 9.17 | 9 | 0.42 | - | 1 | - | **YES** | - | - |
| 1. Soft | -0.54 | 0.07 | -0.71 | 671.19 | 3.55 | 9 | 0.94 | - | - | - | - | - | **YES** |
| 1. Face relaxed | -0.47 | 0.08 | -1.01 | 565.16 | 5.75 | 9 | 0.76 | - | - | - | **YES** | - | - |
| 1. Show expression | -0.44 | 0.08 | 1.12 | 565.16 | 14.41 | 9 | 0.11 | - | - | - | **YES** | - | - |
| 1. Age | -0.41 | 0.07 | -0.97 | 671.19 | 2.50 | 9 | 0.98 | - | 2 | - | - | - | - |
| 1. Raise eyebrows | -0.29 | 0.08 | 2.71 | 565.16 | 17.83 | 9 | 0.04 | - | - | - | **YES** | - | - |
| 1. Healthy | -0.20 | 0.07 | -2.88 | 671.19 | 8.75 | 9 | 0.46 | 1,2,3 | - | - | - | **YES** | - |
| 1. Young | -0.19 | 0.07 | -3.87 | 671.19 | 9.54 | 9 | 0.39 | 1,2 | - | - | - | - | - |
| 1. Fresh | -0.14 | 0.07 | -6.32 | 671.19 | 18.53 | 9 | 0.03 | - | - | - | - | **YES** | - |
| 1. Full | -0.14 | 0.07 | -1.90 | 671.19 | 10.64 | 9 | 0.30 | 1 | - | - | - | - | **YES** |
| 1. Lifted | -0.12 | 0.07 | 0.53 | 671.19 | 9.77 | 9 | 0.37 | - | - | 2,3 | - | - | **YES** |
| 1. Tight | -0.09 | 0.07 | 0.74 | 671.19 | 9.11 | 9 | 0.43 | 1,2,3 | - | - | - | - | **YES** |
| 1. Overall quality | -0.08 | 0.07 | -4.61 | 671.19 | 10.93 | 9 | 0.28 | - | - | - | - | - | - |
| 1. Squint | -0.07 | 0.08 | -1.40 | 565.16 | 2.60 | 9 | 0.98 | 2 | - | - | **YES** | - | - |
| 1. Elasticity | -0.04 | 0.07 | 0.09 | 671.19 | 9.23 | 9 | 0.42 | 1,2,3 | - | - | - | - | **YES** |
| 1. Firm | 0.01 | 0.07 | -1.46 | 671.19 | 3.95 | 9 | 0.91 | 1,3 | - | - | - | - | **YES** |
| 1. Youthful | 0.05 | 0.07 | -4.12 | 671.19 | 8.84 | 9 | 0.45 | 1,3 | - | - | - | **YES** | - |
| 1. Photos | 0.05 | 0.07 | -0.07 | 671.19 | 3.17 | 9 | 0.96 | - | - | - | - | - | - |
| 1. Rested | 0.11 | 0.08 | -3.25 | 565.16 | 11.84 | 9 | 0.22 | - | - |  | - | **-** | - |
| 1. Good | 0.12 | 0.07 | -6.00 | 671.19 | 21.74 | 9 | 0.01 | - | - |  | - | **YES** | - |
| 1. Bright | 0.13 | 0.07 | -3.61 | 671.19 | 7.10 | 9 | 0.63 | 1,3 | 1 |  | - | **YES** | - |
| 1. Frown | 0.15 | 0.07 | -0.74 | 565.16 | 5.26 | 9 | 0.81 | - | - |  | **YES** | - | - |
| 1. Angles | 0.16 | 0.07 | -2.72 | 671.19 | 6.21 | 9 | 0.72 | - | - |  | - | - | - |
| 1. Smooth | 0.22 | 0.07 | 1.33 | 671.19 | 8.11 | 9 | 0.52 | 3 | - |  | - | - | **YES** |
| 1. Vibrant | 0.24 | 0.07 | -5.75 | 671.19 | 14.39 | 9 | 0.11 | - | - |  | - | **YES** | - |
| 1. Attractive | 0.26 | 0.07 | -1.79 | 671.19 | 7.96 | 9 | 0.54 | - | - |  | - | - | - |
| 1. Rejuvenated | 0.27 | 0.07 | -5.88 | 671.19 | 16.41 | 9 | 0.06 | - | - |  | - | **YES** | - |
| 1. Tone (color) | 0.28 | 0.07 | 1.77 | 671.19 | 11.20 | 9 | 0.26 | - | - | - | - | - | - |
| 1. On screen | 0.32 | 0.07 | 1.87 | 540.84 | 19.12 | 9 | 0.02 | 2 | - | - | - | - | - |
| 1. Hydrated | 0.34 | 0.07 | 3.19 | 671.19 | 12.51 | 9 | 0.19 | - | - | - | - | - | **YES** |
| 1. Prolife | 0.34 | 0.07 | -0.75 | 671.19 | 5.14 | 9 | 0.82 | - | - | - | - | - | - |
| 1. Without makeup | 0.36 | 0.07 | -0.57 | 565.16 | 7.24 | 9 | 0.61 | - | 3 | - | - | - | - |
| 1. Glow | 0.37 | 0.07 | -2.86 | 671.19 | 7.91 | 9 | 0.54 | - | - | - | - | **YES** | - |
| 1. Even tone | 0.37 | 0.07 | 2.11 | 671.19 | 11.88 | 9 | 0.22 | - | - | - | - | - | **YES** |
| 1. Texture | 0.46 | 0.07 | 2.61 | 671.19 | 9.16 | 9 | 0.42 | 1,2,3 | - | - | - | - | **YES** |
| 1. Refreshed | 0.52 | 0.07 | -3.25 | 605.05 | 12.81 | 9 | 0.17 | - | - | - | - | **-** | - |
| 1. Radiant | 0.73 | 0.07 | -2.18 | 671.19 | 13.84 | 9 | 0.13 | - | - | - | - | **YES** | - |
| 1. Up close | 1.07 | 0.06 | -0.71 | 671.19 | 7.41 | 9 | 0.59 | - | - | - | - | - | - |
| 1. Bright light | 1.15 | 0.06 | -1.70 | 671.19 | 9.43 | 9 | 0.40 | - | 1,3 | - | - | - | - |
| 1. Flawless | 1.31 | 0.07 | -1.75 | 671.19 | 12.91 | 9 | 0.17 |  |  | - | - | - | **YES** |

| **Scales** | **Item Fit Statistics** | | | | | | | **Differential Item Function*** | | | **Short Forms** | |
| --- | --- | --- | --- | --- | --- | --- | --- | --- | --- | --- | --- | --- |
| **SKIN FEELS** | **Location** | **SE** | **Fit**  **Residual** | **DF** | **χ^2^** | **DF** | **p-value** | **Age** | **Gender** | **Body Face** | **Skin Rejuvenation** | **Skin Quality** |
| 1. Natural | -1.37 | 0.08 | 1.37 | 629.75 | 9.31 | 8 | 0.32 | - | - | - | - | - |
| 1. Clean | -0.71 | 0.07 | 1.37 | 629.75 | 14.98 | 8 | 0.06 | 1,2,3 | - | - | - | - |
| 1. Thick | -0.68 | 0.07 | 2.47 | 629.75 | 8.46 | 8 | 0.39 | - | - | 3 | - | **YES** |
| 1. Comfortable | -0.57 | 0.07 | 1.18 | 629.75 | 7.12 | 8 | 0.52 | - | - | - | - | - |
| 1. Soft | -0.41 | 0.07 | -2.00 | 629.75 | 7.68 | 8 | 0.47 | - | - | - | - | **YES** |
| 1. Healthy | -0.39 | 0.07 | -4.17 | 629.75 | 14.91 | 8 | 0.06 | - | - | - | **YES** | - |
| 1. Good | -0.22 | 0.07 | -5.40 | 629.75 | 23.06 | 8 | 0.00 | 3 | - | - | **YES** | **-** |
| 1. Tight | -0.12 | 0.07 | 1.25 | 629.75 | 7.81 | 8 | 0.45 | 1,2,3 | 2 | 2 | - | **YES** |
| 1. Firm | 0.01 | 0.07 | 0.22 | 629.75 | 1.54 | 8 | 0.99 | 1,2,3 | - | - | - | **YES** |
| 1. Full | 0.02 | 0.07 | -0.58 | 629.75 | 7.21 | 8 | 0.51 | 1,2,3 | - | - | - | **YES** |
| 1. Smooth | 0.11 | 0.07 | -1.41 | 629.75 | 1.77 | 8 | 0.99 | 1,2,3 | - | - | - | **YES** |
| 1. Youthful | 0.16 | 0.07 | -3.18 | 629.75 | 8.25 | 8 | 0.41 | 1,2,3 | - | - | **YES** | - |
| 1. Refreshed | 0.18 | 0.07 | -4.78 | 629.75 | 11.24 | 8 | 0.19 | - | - | - | **YES** | **-** |
| 1. Elasticity | 0.18 | 0.07 | 0.09 | 629.75 | 3.13 | 8 | 0.93 | 1,2,3 | - | - | **-** | **YES** |
| 1. Rejuvenated | 0.38 | 0.07 | -5.24 | 629.75 | 14.23 | 8 | 0.08 | - | - | - | **YES** | - |
| 1. Hydrated | 0.41 | 0.07 | 2.90 | 629.75 | 14.15 | 8 | 0.08 | 2 | - | - | - | **YES** |
| 1. Clear | 0.53 | 0.07 | 1.76 | 629.75 | 8.70 | 8 | 0.37 | 3 | - | - | - | - |
| 1. Texture | 0.59 | 0.07 | -1.42 | 629.75 | 5.78 | 8 | 0.67 | 1,2,3 | - | - | - | **YES** |
| 1. New | 0.60 | 0.07 | -3.84 | 629.75 | 14.20 | 8 | 0.08 | - | - | - | **YES** | - |
| 1. Flawless | 1.31 | 0.07 | -0.44 | 629.75 | 13.37 | 8 | 0.10 | - | 2 | - | - | **-** |

For DIF analysis: 1 = random sample 1, 2 = random sample 2, 3 = random sample 3
