# Supplementary figures and images for: The SKIN-Q: An Innovative Patient-Reported Outcome Measure for Evaluating Minimally Invasive Skin Treatments for the Face and Body
Source: Facial Plast Surg Aesthet Med. 2024 Jun 6;26(3):247–55. doi: 10.1089/fpsam.2023.0204 (PMC11295662; doi:10.1089/fpsam.2023.0204)

| **S6a: Threshold map for how skin looks** |
| --- |
| 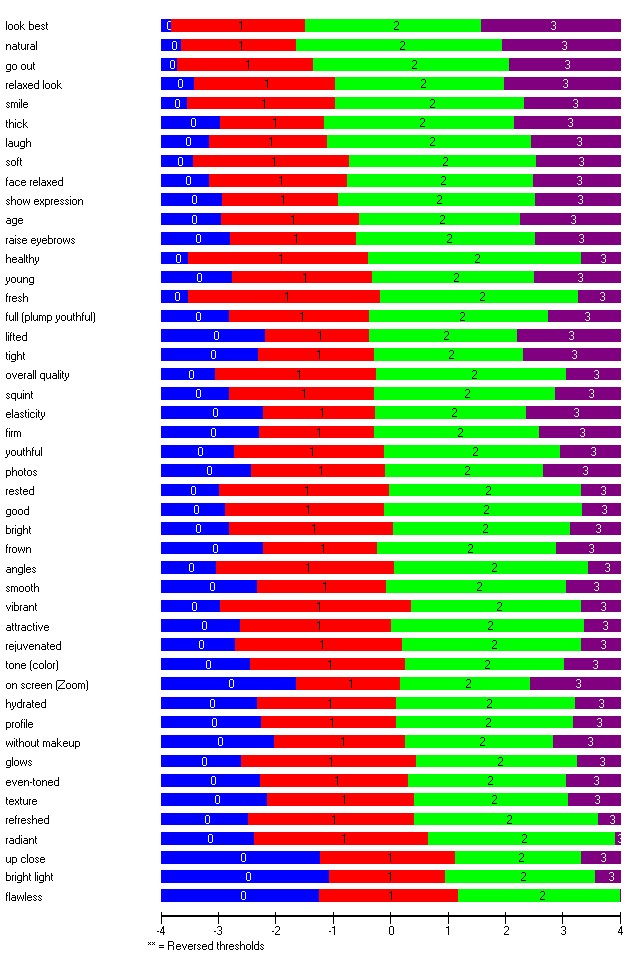 |

Supplement: Supplementary Data S6a [file fpsam.2023.0204_suppl_datas6a.docx]

| **S6b: Person-item threshold distribution for how skin looks by face and body.** |
| --- |
| 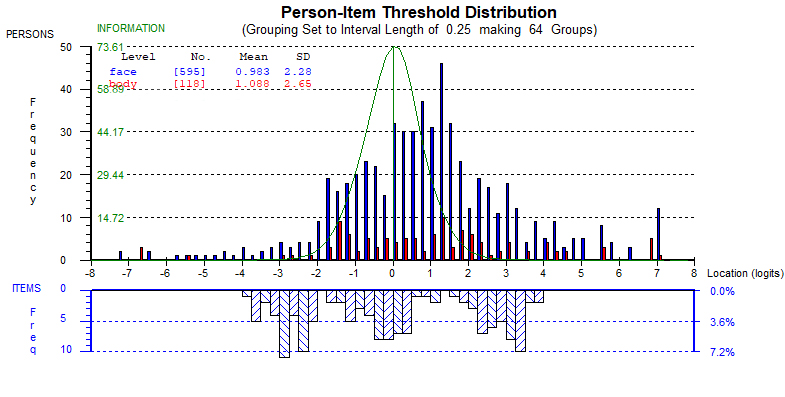 |

Supplement: Supplementary Data S6b [file fpsam.2023.0204_suppl_datas6b.docx]

| **S7a: Threshold map for how skin feels** |
| --- |
| 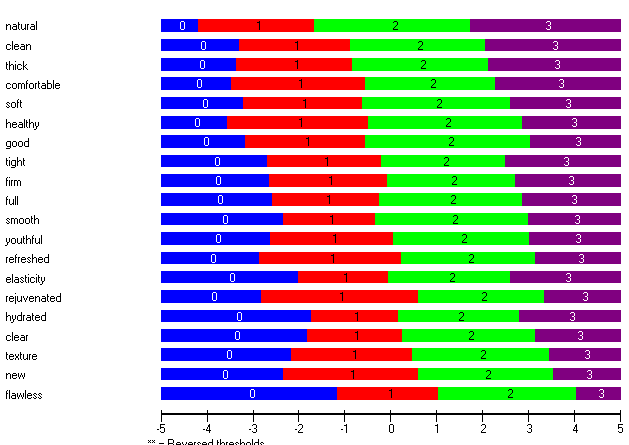 |

Supplement: Supplementary Data S7a [file fpsam.2023.0204_suppl_datas7a.docx]

| **S7b: Person-item threshold distribution for how skin feels** |
| --- |
| 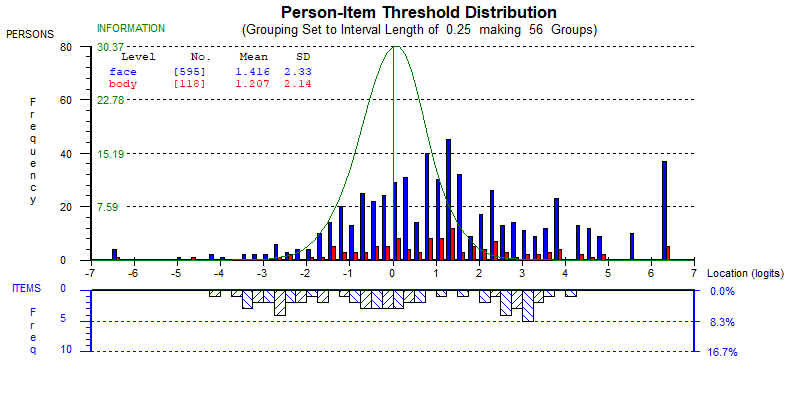 |

Supplement: Supplementary Data S7b [file fpsam.2023.0204_suppl_datas7b.docx]
