# Supplementary material for: The SKIN-Q: An Innovative Patient-Reported Outcome Measure for Evaluating Minimally Invasive Skin Treatments for the Face and Body
Source: Facial Plast Surg Aesthet Med. 2024 Jun 6;26(3):247–55. doi: 10.1089/fpsam.2023.0204 (PMC11295662; doi:10.1089/fpsam.2023.0204)
Supplement: Supplementary Data S8 [file fpsam.2023.0204_suppl_datas8.docx]

**S8: SKIN-Q mean scores by severity of facial lines**

| DOMAIN | SCALE |  | None | | | Mild | | | Moderate | | | Severe/Very Severe | | | P-value |
| --- | --- | --- | --- | --- | --- | --- | --- | --- | --- | --- | --- | --- | --- | --- | --- |
|  |  |  | N | Mean | SD | N | Mean | SD | N | Mean | SD | N | Mean | SD |  |
| SKIN LOOKS | ITEM LIBRARY | Forehead lines | 89 | 62 | 14 | 191 | 57 | 14 | 137 | 52 | 14 | 55 | 46 | 15 | <0.001 |
|  |  | Glabellar lines | 111 | 61 | 14 | 185 | 56 | 14 | 120 | 53 | 15 | 56 | 48 | 15 | <0.001 |
|  |  | Crow’s feet | 147 | 60 | 14 | 209 | 56 | 14 | 85 | 50 | 15 | 31 | 44 | 13 | <0.001 |
|  |  | Nasolabial folds | 158 | 62 | 14 | 179 | 55 | 14 | 110 | 51 | 13 | 25 | 39 | 15 | <0.001 |
|  |  | Lip lines | 302 | 59 | 14 | 109 | 51 | 12 | 47 | 46 | 15 | 14 | 39 | 13 | <0.001 |
|  |  | Marionette lines | 240 | 60 | 14 | 131 | 54 | 13 | 69 | 51 | 12 | 32 | 40 | 16 | <0.001 |
|  | SKIN REJUVENATION | Forehead lines | 89 | 61 | 15 | 191 | 58 | 16 | 137 | 52 | 16 | 55 | 46 | 18 | <0.001 |
|  |  | Glabellar lines | 111 | 60 | 16 | 185 | 56 | 16 | 120 | 53 | 16 | 56 | 48 | 17 | <0.001 |
|  |  | Crow’s feet | 147 | 60 | 16 | 209 | 56 | 16 | 85 | 50 | 17 | 31 | 45 | 16 | <0.001 |
|  |  | Nasolabial folds | 158 | 61 | 16 | 179 | 55 | 15 | 110 | 51 | 16 | 25 | 39 | 16 | <0.001 |
|  |  | Lip lines | 302 | 59 | 16 | 109 | 51 | 15 | 47 | 46 | 17 | 14 | 38 | 15 | <0.001 |
|  |  | Marionette lines | 240 | 59 | 16 | 131 | 54 | 16 | 69 | 51 | 14 | 32 | 39 | 18 | <0.001 |
|  | SKIN QUALITY | Forehead lines | 89 | 61 | 13 | 191 | 57 | 14 | 137 | 53 | 13 | 55 | 47 | 15 | <0.001 |
|  |  | Glabellar lines | 111 | 61 | 14 | 185 | 56 | 14 | 120 | 53 | 14 | 56 | 48 | 13 | <0.001 |
|  |  | Crow’s feet | 147 | 61 | 13 | 209 | 55 | 14 | 85 | 50 | 14 | 31 | 44 | 12 | <0.001 |
|  |  | Nasolabial folds | 158 | 62 | 14 | 179 | 55 | 13 | 110 | 51 | 13 | 25 | 41 | 14 | <0.001 |
|  |  | Lip lines | 302 | 59 | 14 | 109 | 51 | 11 | 47 | 47 | 14 | 14 | 39 | 14 | <0.001 |
|  |  | Marionette lines | 240 | 60 | 14 | 131 | 54 | 12 | 69 | 50 | 11 | 32 | 38 | 15 | <0.001 |
|  | FACIAL MOVEMENT | Forehead lines | 89 | 62 | 15 | 191 | 58 | 14 | 137 | 50 | 14 | 55 | 44 | 15 | <0.001 |
|  |  | Glabellar lines | 111 | 61 | 15 | 185 | 56 | 14 | 120 | 52 | 16 | 56 | 47 | 14 | <0.001 |
|  |  | Crow’s feet | 147 | 61 | 15 | 209 | 55 | 14 | 85 | 48 | 14 | 31 | 42 | 14 | <0.001 |
|  |  | Nasolabial folds | 158 | 62 | 15 | 179 | 54 | 13 | 110 | 50 | 12 | 25 | 37 | 13 | <0.001 |
|  |  | Lip lines | 302 | 59 | 15 | 109 | 49 | 13 | 47 | 45 | 15 | 14 | 42 | 11 | <0.001 |
|  |  | Marionette lines | 240 | 60 | 15 | 131 | 51 | 13 | 69 | 50 | 13 | 32 | 41 | 14 | <0.001 |
| SKIN FEELS | ITEM LIBRARY | Forehead lines | 89 | 66 | 19 | 191 | 63 | 18 | 137 | 58 | 17 | 55 | 50 | 17 | <0.001 |
|  |  | Glabellar lines | 111 | 65 | 20 | 185 | 61 | 18 | 120 | 58 | 18 | 56 | 55 | 18 | 0.003 |
|  |  | Crow’s feet | 147 | 66 | 18 | 209 | 60 | 18 | 85 | 56 | 18 | 31 | 50 | 16 | <0.001 |
|  |  | Nasolabial folds | 158 | 67 | 20 | 179 | 60 | 17 | 110 | 55 | 15 | 25 | 47 | 17 | <0.001 |
|  |  | Lip lines | 302 | 65 | 19 | 109 | 56 | 16 | 47 | 51 | 14 | 14 | 43 | 16 | <0.001 |
|  |  | Marionette lines | 240 | 66 | 19 | 131 | 58 | 17 | 69 | 54 | 14 | 32 | 47 | 17 | <0.001 |
|  | SKIN REJUVENATION | Forehead lines | 89 | 65 | 19 | 191 | 62 | 19 | 137 | 58 | 18 | 55 | 49 | 20 | <0.001 |
|  |  | Glabellar lines | 111 | 64 | 19 | 185 | 60 | 19 | 120 | 58 | 19 | 56 | 55 | 20 | 0.026 |
|  |  | Crow’s feet | 147 | 64 | 19 | 209 | 60 | 19 | 85 | 55 | 19 | 31 | 50 | 19 | <0.001 |
|  |  | Nasolabial folds | 158 | 66 | 20 | 179 | 59 | 18 | 110 | 55 | 17 | 25 | 46 | 19 | <0.001 |
|  |  | Lip lines | 302 | 64 | 19 | 109 | 55 | 18 | 47 | 50 | 16 | 14 | 42 | 19 | <0.001 |
|  |  | Marionette lines | 240 | 64 | 19 | 131 | 58 | 18 | 69 | 54 | 16 | 32 | 46 | 19 | <0.001 |
|  | SKIN QUALITY | Forehead lines | 89 | 66 | 17 | 191 | 62 | 17 | 137 | 58 | 16 | 55 | 49 | 17 | <0.001 |
|  |  | Glabellar lines | 111 | 65 | 17 | 185 | 61 | 17 | 120 | 58 | 17 | 56 | 54 | 17 | <0.001 |
|  |  | Crow’s feet | 147 | 66 | 16 | 209 | 60 | 17 | 85 | 56 | 17 | 31 | 49 | 16 | <0.001 |
|  |  | Nasolabial folds | 158 | 67 | 17 | 179 | 60 | 16 | 110 | 55 | 14 | 25 | 47 | 18 | <0.001 |
|  |  | Lip lines | 302 | 64 | 16 | 109 | 56 | 16 | 47 | 51 | 15 | 14 | 40 | 16 | <0.001 |
|  |  | Marionette lines | 240 | 65 | 17 | 131 | 58 | 16 | 69 | 54 | 14 | 32 | 46 | 17 | <0.001 |

All p-values for non-parametric the same as ANOVA
